# Changes in the characteristics of patients with latex allergy from 1999 to 2014

**DOI:** 10.20407/fmj.2019-013

**Published:** 2020-02-11

**Authors:** Manabu Kawai, Yasuto Kondo, Yoichi Nakajima, Ikuya Tsuge, Tetsushi Yoshikawa, Akiko Yagami, Michiko Aihara, Zenro Ikezawa, Yukihiro Ohya, Taeru Kitabayashi, Hirohisa Saito, Rumiko Shibata, Toru Naito, Susumu Harada, Michihiro Hide, Kayoko Matsunaga, Katsuyuki Miyasaka, Akira Akasawa

**Affiliations:** 1 Department of Pediatrics, Fujita Health University, School of Medicine, Toyoake, Aichi, Japan; 2 Department of Pediatrics, Fujita Health University Bantane Hospital, Nagoya, Aichi, Japan; 3 Department of Pediatrics, Yachiyo Hospital, Anjo, Aichi, Japan; 4 Department of Dermatology, Fujita Health University Bantane Hospital, Nagoya, Aichi, Japan; 5 Department of Dermatology, Yokohama City University School of Medicine, Yokohama, Kanagawa, Japan; 6 Ai Derma & Allergy, Yokohama, Kanagawa, Japan; 7 National Center for Child Health and Development, Setagaya, Tokyo, Japan; 8 Department of Pediatrics, International University of Health and Welfare Mita Hospital, Minato, Tokyo, Japan; 9 Department of Allergy & Immunology, National Center for Child Health and Development, Setagaya, Tokyo, Japan; 10 Department of Pediatrics, Fukuoka National Hospital, Fukuoka, Fukuoka, Japan; 11 Department of General Dentistry, Fukuoka Dental College, Fukuoka, Fukuoka, Japan; 12 Harada Dermatology Clinic, Nishinomiya, Hyogo, Japan; 13 Department of Dermatology, Hiroshima University School of Medicine, Hiroshima, Hiroshima, Japan; 14 Department of Integrative Medical Science for Allergic Disease, Fujita Health University, School of Medicine, Toyoake, Aichi, Japan; 15 St. Luke’s International University, Chuo, Tokyo, Japan; 16 Nasunogahara Clinic, Otawara, Tochigi, Japan

**Keywords:** Latex allergy, Type I allergy, Health care workers

## Abstract

**Objectives:**

We conducted a multicenter study using the same questionnaire in 1999 and 2014 to investigate changes in the characteristics of patients with latex allergy.

**Methods:**

We mailed questionnaires on latex allergy to hospitals in Japan that were members of the Japanese Latex Allergy Society.

**Results:**

We compared the 25 responses received in 2014 and the 81 responses received in 1999. With regard to the age distribution, the number of patients with latex allergy in their 20s declined significantly from 1999 to 2014 (P=0.004). The largest proportion of latex allergy cases was observed among those aged <10 years. The incidence of cases caused by medical rubber gloves decreased significantly (P=0.004). Moreover, latex-fruit syndrome increased from 15% to 40% (P=0.006).

**Conclusions:**

Our findings indicate that the frequency of occurrence of latex allergy in people in their 20s decreased from 1999 to 2014. The largest proportion of latex allergy cases was observed among those aged <10 years. Future measures to protect children are required.

## Introduction

Latex allergy causes immediate type I allergic reactions induced by the interaction between water-soluble protein antigens contained in natural rubber latex and antigen-specific immunoglobulin E antibodies in the patient’s blood. Exposure to products containing natural rubber latex can cause various allergic symptoms including urticaria, asthma-like symptoms, and anaphylaxis.^1^

The mechanism of latex allergen exposure includes direct contact and the inhalation of powder found in powdered natural rubber gloves.^2^ Previously reported risk factors for latex-related allergic reactions include atopic dermatitis and repeated medical procedures.^3,4^ In addition, latex allergy exhibits cross-reactivity with fruit allergies in 30%–50% of cases, particularly for high-risk foods including bananas, kiwi fruits, and avocados.^5^

The existence of latex allergy was first reported in 1979 by Nutter.^6^ In the 1980s, rubber gloves were increasingly used to prevent the spread of diseases such as hepatitis B and human immunodeficiency virus. At the same time, the number of patients with latex allergy increased, particularly among health care workers who frequently used rubber gloves.^7^

In 1991, the United States Food and Drug Administration (FDA) initiated activities to raise awareness about latex allergy in the *Medical Bulletin*, considering the reports of > 1,000 cases of latex-related anaphylaxis and 15 deaths caused by anaphylactic shock. In 1999, the FDA proposed the *Medical Glove Guidance Manual*, after which latex-free and powder-free gloves were introduced.^8^

In Japan, awareness was raised regarding anaphylactic reactions caused by medical products made from natural rubber, such as surgical gloves, in 1992. However, the recognition of potential latex-related allergic reactions was low, and available countermeasures were inadequate. Therefore, the Japanese Latex Allergy Society was formed in 1996 to address these problems. The Latex Allergy Forum, held in 1998, aimed to raise awareness regarding this condition. In 1999, package inserts for medical devices made from latex were revised, and the appropriate labeling of any product containing latex was made mandatory.

The onset frequency of latex allergy ranged from 2.9% to 12.1% in an epidemiological survey conducted among health care workers in Europe and the United States in the 1990s.^9–14^ Another epidemiological survey of latex allergy conducted from 2000 onward among outpatients at a teaching hospital in Denmark reported sensitization rates of 6.1% from 2002 to 2005 and 1.2% from 2010 to 2013, indicating a significant decrease over time (*P*<0.0001).^15^

In Japan, an epidemiological survey conducted by Kano et al. on the topic of latex allergy among health care workers at teaching hospitals reported the frequency of onset as 6.8% in 1997 and 3.3% in 2004.^16^ Although previous studies have reported a decrease over time in the number of cases has been reported, these studies were single-center investigations, and it is possible that differences among hospitals on characteristics such as the center’s size, patient background, and latex countermeasures may have influenced the results. Therefore, conducting a comparative investigation of multiple centers would facilitate a more accurate evaluation regarding the current scenario of changes in the incidence of latex allergy.

In 1999, we conducted a questionnaire survey investigating latex allergy in Japan.^17^ We then repeated this survey using the same questionnaire to investigate the changes in latex allergy over 15 years. In 1999, our survey results indicated that numerous patients with latex allergy were in their 20s and 30s and that the most common cause of the allergy was exposure to medical rubber gloves. Moreover, latex-fruit syndrome was found in 15% of the cases of latex allergy.

## Methods

In the present study, we compared the results of the survey conducted in 2014 with those of the original investigation conducted in 1999 using the same questionnaire. For the 2014 survey, we mailed questionnaires to hospitals in Japan that were members of the Japanese Latex Allergy Society. We instructed these hospitals to provide information regarding their patients with latex allergy who were currently undergoing treatment and requested that the questionnaires be mailed back after completion.

The survey questions (Table 1) included patient information such as age, sex, risk factors, method of diagnosis, cause of symptoms, induced symptoms, other allergic disease complications, and, when latex-fruit syndrome was present, foods causing symptoms.

This study was approved by the Medical Research Ethics Committee of Fujita Health University (HM15-025).

### Statistical analysis

We compared the results from the 1999 and 2014 surveys using chi-squared tests calculated with Prism 6 (GraphPad Software Inc.). The level of significance was set at *P*<0.05.

## Results

The survey conducted in 1999 targeted 13 centers and received responses pertaining to 81 patients with latex allergy. In comparison, the survey conducted in 2014 targeted 12 centers and received responses pertaining to 25 patients with latex allergy.

**Figure 1:**
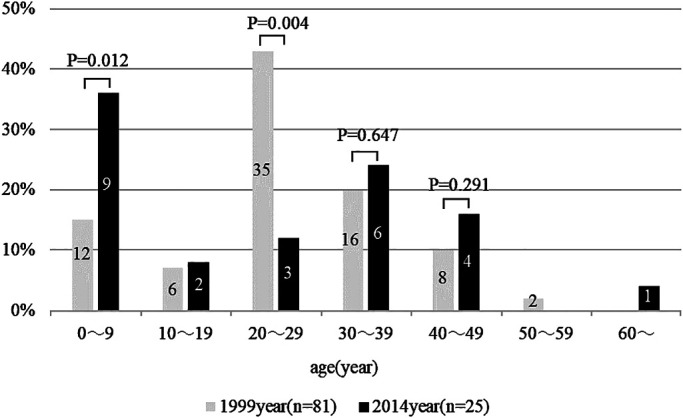
Age distribution of patients with latex allergy in 1999 and 2014 The bars in the graph indicate the numbers of patients. In the present study, the number of patients in their 20s decreased significantly, whereas no changes were observed for the numbers of patients in their 30s or 40s. Patients aged <10 years made up the largest proportion of cases overall.

The 1999 survey collected data on 23 male patients and 58 female patients, whereas the 2014 survey collected data on 9 male patients and 16 female patients. In terms of the age distribution, the number of patients in their 20s was lower in the 2014 survey than in the 1999 survey (*P*=0.004). No significant changes over time were observed in the numbers of patients in their 30s (*P*=0.647) or 40s (*P*=0.291) (Figure 1). Overall, those aged <10 years accounted for the largest proportion of the study population.

**Figure 2:**
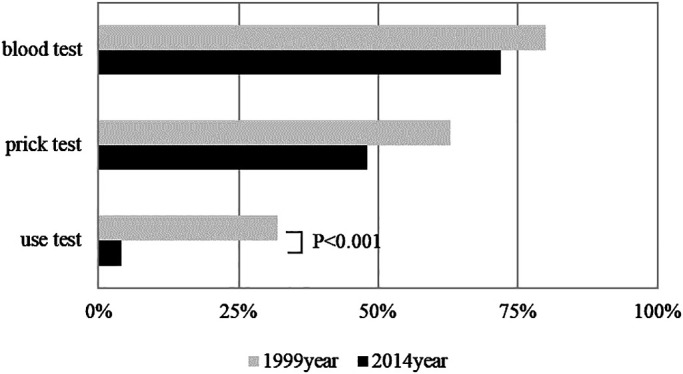
Methods for the diagnosis of latex allergy in 1999 and 2014 Although no changes were observed in the implementation rates for blood or prick tests, the use test implementation rate decreased significantly.

Table 2 shows the grounds for diagnosis in the 2014 survey, and Figure 2 presents a comparison of the diagnostic methods reported in the two surveys. Although no changes were observed in the rates of implementation of blood tests or prick tests, the implementation rates of use tests decreased significantly over time.

**Figure 3:**
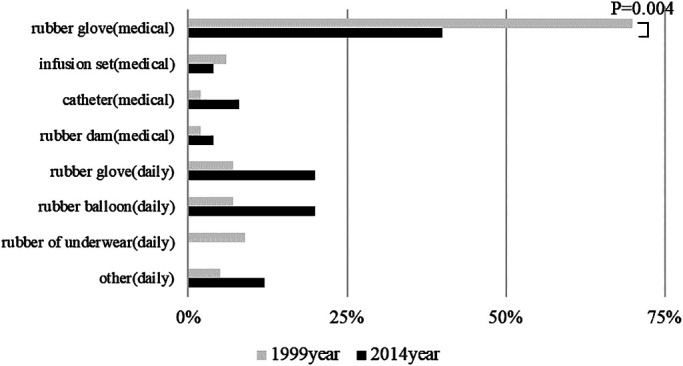
Symptom-causing latex products in 1999 and 2014 The number of cases caused by medical rubber gloves decreased significantly. However, no significant changes were observed for the numbers of cases caused by other latex products.

**Figure 4:**
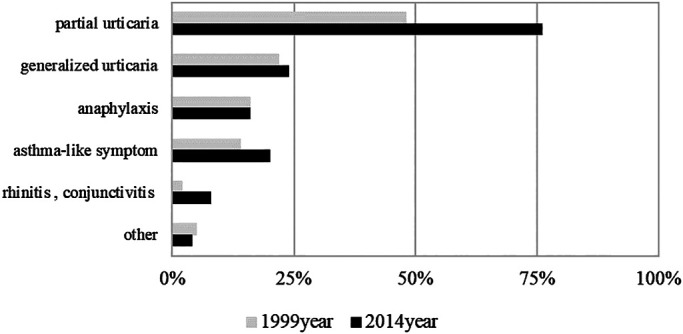
Comparison of symptoms resulting from coming into contact with latex products in 1999 and 2014 Across all types of induced symptoms, no significant changes were noted in the onset rate.

In both surveys, the most common cause of latex allergy symptoms was exposure to medical rubber gloves. The number of cases of latex allergy onset significantly decreased from 1999 to 2014 (*P*=0.004) (Figure 3). Hardly any changes in the onset rate of induced symptoms were noted between the two surveys (Figure 4).

**Figure 5:**
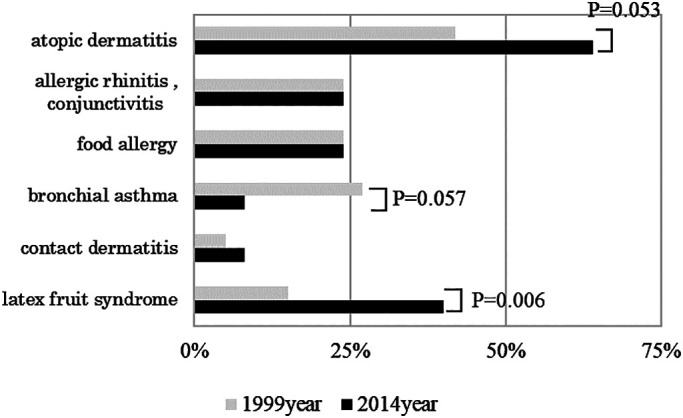
Complications of patients with latex allergy in 1999 and 2014 Although no significant changes were observed, atopic dermatitis showed an increasing trend, and bronchial asthma showed a decreasing trend. Further, the proportion of cases where latex-fruit syndrome was present showed a significant increase over the study period.

In terms of complications, the percentage of patients with latex allergy who had latex-fruit syndrome increased from 15% in 1999 to 40% in 2014 (*P*=0.006) (Figure 5). A large number of patients were affected by fruits typically considered high risk, including bananas (five cases), kiwi fruit (four cases), and avocado (three cases). Reactions to other foods, including cherries (two cases), peaches (one case), carrots (one case), tomatoes (one case), walnuts (one case), loquats (one case), and pears (one case), were also observed.

## Discussion

In 1999, a law was established in Japan that made labeling mandatory for medical products containing latex, and the protocols for package inserts for medical products made using natural rubber were revised. As a result, it is no longer as difficult as it once was to determine which medical devices and equipment contain natural rubber, making it easier to create completely latex-free environments, with no products made from natural rubber. Further, powder-free gloves are increasingly being used.

Although it was impossible to compare all parameters in the present study because of missing background data on some patients, we observed that almost all patients with latex allergy in 1999 were nurses, whereas the percentage of patients with latex allergy who were nurses was only 16% in 2014 (data not shown). Likewise, a previous investigation targeting a university hospital in Maryland reported that the introduction of latex-free and powder-free gloves reduced the rate of latex allergy symptom onset from 42% to 29% among health care workers.^18^

These social background factors as well as the decrease in the proportion of nurses among patients with latex allergy suggest that the number of new cases of latex allergy symptom onset caused by exposure to medical rubber gloves declined among individuals in their 20s. Of the examined age groups, the age group of <10 years made up the largest proportion of patients with latex-related allergic reactions. This finding may be attributable to the decrease in patients in their 20s with latex allergy, leading to an increase in the proportion of patients with latex allergy who were aged <10 years. Although the implementation of countermeasures against the use of medical rubber gloves has enabled the prevention of the onset of latex allergy among health care workers, further action is required to prevent latex allergy onset among children.

The significant decline in the use test implementation rate from the 1999 survey to the 2014 survey can be attributed to the difficulty of obtaining gloves that contain large amounts of latex protein in Japan in later years.

The number of cases of symptom onset associated with medical rubber glove exposure may have decreased because the implementation of countermeasures against the use of medical rubber gloves reduced the proportion of patients who were nurses. However, no changes were noted in the number of cases resulting from the use of other medical equipment or everyday rubber products. This indicates that an increase in awareness regarding the use of latex products besides medical rubber gloves is still required.

In the present survey, the investigation of the allergic disease comorbidity rate revealed that atopic dermatitis is experiencing an increasing trend, bronchial asthma is experiencing a decreasing trend, and the trends of allergic rhinitis and food allergies have both leveled off. There were no reports of epidemiological trends targeting the general population in Japan. These trends targeting elementary school children in western Japan was conducted in 1992, 2002, and 2012.^19^ This previous study found that atopic dermatitis decreased (from 17.27% in 1992 to 13.81% in 2002 and 11.72% in 2012), allergic rhinitis increased (from 15.89% in 1992 to 20.45% in 2002 and 28.05% in 2012), and bronchial asthma leveled off (4.60% in 1992, 6.54% in 2002, and 4.73% in 2012). Food allergies, which were investigated only in 2012, were found in 3.56% of the study sample. Differences in these epidemiological trends between our results and the findings of this previous study suggest that specific changes may occur in patients with latex allergy. Atopic dermatitis is a high-risk factor for latex allergy. The present survey indicated a high frequency of comorbidities and an increasing trend.

Additionally, our findings showed that the latex-fruit syndrome comorbidity rate increased from 15% in 1999 to 40% in 2014. In a large number of patients in the present study, this reaction was observed to bananas, kiwi fruits, or avocados, which was consistent with previously reported results.^5^ Cross-reactivity occurs between pollen and fruit.^20^ Therefore, the previously reported increase in pollinosis cases^21^ may have resulted in the observed increase in fruit allergy cases. However, in the present study, no changes were noted in the pollinosis comorbidity rates from 1999 to 2014. It is possible that the recognition of latex-fruit syndrome has increased in recent years. In the future, mid- and long-term investigations are required to clarify the trend in latex-fruit syndrome comorbidity.

### Limitations

Because responses were not received from the same hospitals in 1999 and 2014, we were unable to fully investigate to the extent of the decrease in latex allergy. Further, the 2014 survey did not investigate the countermeasures against latex allergy implemented over the past 15 years. Therefore, we were unable to test whether there were differences in the risk of latex allergy onset between hospitals that had implemented these countermeasures and those that had not.

## Conclusions

This multicenter investigation of changes in latex allergy onset from 1999 to 2014 revealed that, during these years, latex allergy onset decreased among patients in their 20s, and the onset rate was highest among patients aged <10 years. Although the incidence of cases caused by medical rubber gloves significantly decreased between the two surveys, no changes were observed in the number of cases of onset resulting from other latex products. During the same time period, latex-fruit syndrome comorbidity rates increased.

Previously, latex allergy symptoms commonly occurred among health care workers. The subsequent implementation of countermeasures resulted in a decrease in the frequency of latex allergy onset in this group. However, countermeasures for other patients who frequently come into contact with products containing natural rubber, such as children, appear to be inadequate. Action should be taken to prevent or reduce latex allergy onset among children. An increase in awareness regarding the subject of latex allergy should be further promoted because latex-related allergic reactions are still incompletely controlled. Additional surveys, such as the 2014 survey described in this article, should be conducted in the future.

## Figures and Tables

**Table1:** Questionnaire on the topic of latex allergy

1. age	( )
2. sex	( )
3. risk factor	please choose a number from the following choices
	(1) doctor
	(2) nurse
	(3) laboratory technician
	(4) dentist
	(5) dental assistant
	(6) dental hygienist
	(7) other medical staff
	(8) student
	(9) other occupations dealing with natural rubber
	(10) atopic dermatitis
	(11) repeat medical procedure
	(12) other
4. diagnostic method	please choose a number from the following choices (multiple answers allowed)
	(1) immediate medical history that is not anaphylaxis with latex
	(2) history of anaphylaxis with latex
	(3) serum diagnosis (IgE positive for antigen)
	3a) latex 3b) Hev b 5 3c) Hev b 6
	(4) prick test (skin test with antigen)
	4a) latex 4b) Hev b 5 4c) Hev b 6
	(5) use test
5. latex product causing symptom	please choose a number from the following choices (multiple answers allowed)
(medical)	(1) rubber glove
	(2) infusion set
	(3) catheter
	(4) rubber dam
	(5) other
(daily)	(1) rubber glove
	(2) rubber balloon
	(3) underwear elastic
	(4) other
6. clinical symptom	please choose a number from the following choices (multiple answers allowed)
	(1) partial urticaria
	(2) generalized urticaria
	(3) asthma-like symptom
	(4) rhinitis
	(5) conjunctivitis
	(6) anaphylaxis
	(7) other
7. complication	please choose a number from the following choices (multiple answers allowed)
	(1) atopic dermatitis
	(2) bronchial asthma
	(3) contact dermatitis
	(4) allergic rhinitis
	(5) allergic conjunctivitis
	(6) food allergy
	(7) latex fruit syndrome
8. symptomatic fruit	please choose a number from the following choices (multiple answers allowed)
	(1) banana
	(2) chestnut
	(3) avocado
	(4) kiwi
	(5) other

**Table2:** Grounds for diagnosis (n=25)

medical history only	3
medical history+blood test positive (latex)	6
medical history+blood test positive (latex+Hev b 6)	3
medical history+prick test positive (latex)	1
medical history+prick test positive (latex+Hev b 6)	1
medical history+blood test positive (latex)+prick test positive (latex)	5
medical history+blood test positive (latex)+prick test positive (latex+Hev b6)	1
medical history+blood test positive (latex+Hev b 6)+prick test positive (latex+Hev b6)	3
medical history+blood test positive (Hev b 6)+prick test positive (latex)	1
medical history+use test	1
